# 518. Comparable Outcomes of Remdesivir and Sotrovimab among High-risk Patients with Mild to Moderate COVID-19 During the Omicron BA.1 Surge

**DOI:** 10.1093/ofid/ofad500.587

**Published:** 2023-11-27

**Authors:** Supavit Chesdachai, Christina G Rivera (O'Connor), Kristin Mara, Hiliary R Teaford, Maria Gonzalez Suarez, Jennifer J Larsen, Ravindra Ganesh, Sidna Tulledge-Scheitel, Raymund R Razonable

**Affiliations:** Mayo Clinic, Rochester, MN; Mayo Clinic, Rochester, MN; Mayo Clinic, Rochester, MN; Mayo Clinic, Rochester, MN; Mayo Clinic, Rochester, MN; Mayo Clinic, Rochester, MN; Mayo Clinic, Rochester, MN; Mayo Clinic, Rochester, MN; Mayo Clinic, Rochester, MN

## Abstract

**Background:**

Two independent placebo-controlled clinical trials conducted prior to SARS-CoV-2 Omicron demonstrated that sotrovimab and remdesivir reduced hospitalization among high-risk outpatients with mild to moderate COVID-19. However, their effectiveness have not been directly compared.

**Methods:**

This retrospective study examined all high-risk outpatients with mild to moderate COVID-19 who received either remdesivir or sotrovimab at Mayo Clinic during the Omicron BA.1 surge from January to March 2022. COVID-19-related hospitalization or death within 28 days were compared between the two treatment groups.

**Results:**

Among 3,257 patients, 2,158 received sotrovimab and 1,099 received remdesivir. Patients treated with sotrovimab were younger (median age, 60 vs. 69 years) and had lower comorbidity (Monoclonal Antibody Screening Score, 5 vs. 6; Charlson Comorbidity Index, 3 vs. 4), but were more likely to be immunocompromised (47.3% vs. 30.2%) than remdesivir-treated patients. The majority (89%) had received at least one dose of COVID-19 vaccine (median, 3 doses [IQR 2-3] for both groups). COVID-19-related hospitalization (1.5% and 1.0% in remdesivir and sotrovimab groups, respectively, p=.15) and mortality within 28 days (0.4% in both groups, p=.82) were similarly low. A propensity score weighted analysis demonstrated no significant difference in the rates of COVID-related hospitalization and death within 28 days between the two groups.

**Conclusion:**

This study demonstrated favorable outcomes that were not significantly different between patients treated with remdesivir or sotrovimab. As sotrovimab is no longer authorized, these results support the continued use of remdesivir for treating high-risk patients with COVID-19 during the current Omicron-predominant epoch.

**Disclosures:**

**Christina G. Rivera (O'Connor), Pharm.D**, Gilead Sciences: Advisor/Consultant|Gilead Sciences: Board Member|Gilead Sciences: Grant/Research Support|Gilead Sciences: Honoraria **Maria Gonzalez Suarez, M.D., Ph.D.**, Gilead: Grant/Research Support **Ravindra Ganesh, M.B.B.S., M.D.**, Alpaca Health: Advisor/Consultant **Raymund R. Razonable, MD**, Allovir: Endpoint Adjudication Committee|American Society of Transplantation: Board Member|Gilead: Grant/Research Support|Novartis: DSMB|Regeneron: Grant/Research Support|Roche: Grant/Research Support

